# Prevalence and clinical characteristics of hemorrhoids among adult surgical inpatients at a public teaching hospital in Kabul, Afghanistan

**DOI:** 10.1097/MS9.0000000000005751

**Published:** 2026-10-12

**Authors:** Bashir Ahmad Qudrati, Abdul Waheed Moradi

**Affiliations:** Public Health Faculty, Kabul University of Medical Sciences, Kabul, Afghanistan

**Keywords:** Afghanistan, clinical characteristics, epidemiology, hemorrhoids, prevalence

## Abstract

**Background:**

Hemorrhoids are common benign anorectal disorders characterized by swelling, pain, and rectal bleeding. Limited evidence exists regarding the prevalence and clinical characteristics of hemorrhoids among adult surgical inpatients in Afghanistan.

**Objective:**

This study aimed to determine the hospital-based prevalence of clinically documented hemorrhoids and to describe their clinical characteristics among adult surgical inpatients at a public teaching hospital in Kabul, Afghanistan.

**Methods:**

This retrospective cross-sectional study was conducted using medical records of 209 adult surgical inpatients with a confirmed diagnosis of hemorrhoids at a public teaching hospital in Kabul, Afghanistan, from January through December 2025. Consecutive sampling was used. Descriptive statistics were used to summarize the findings, and all analyses were performed using SPSS, version 29.

**Results:**

Among 3779 adult surgical inpatients, 229 patients had a clinically documented diagnosis of hemorrhoids, corresponding to a hospital-based prevalence of 6.1%. After applying the predefined exclusion criteria, 209 patients were included in the clinical analysis. The mean age was 49.07 ± 18.27 years, while the median age was 50 years (interquartile range: 33.5–65.0 years), with 128 males (61.2%) and 81 females (38.8%). The highest number of patients was in the 30–39-year age group: 64 (30.6%). Rectal bleeding (126, 60.3%) and anal pain (115, 55.0%) were the most common symptoms. Constipation was the most frequently reported factor among patients with hemorrhoids (111, 53.1%), followed by prolonged standing or sitting (103, 49.3%). Internal hemorrhoids were most common, with third-degree internal hemorrhoids observed in 32 of 106 (30.2%) cases of internal hemorrhoids. A significant association was observed between marital status and hemorrhoid type (*P* = 0.028), whereas no significant associations were found between age, gender, or family history and hemorrhoid grade.

**Conclusion:**

Clinically documented hemorrhoids were identified in 6.1% of adult surgical admissions, representing a hospital-based prevalence within this surgical inpatient population. Rectal bleeding and anal pain were the most common symptoms, while constipation was the most frequently reported factor among patients with hemorrhoids.

## Introduction

Hemorrhoids, or piles, are among the most common benign anorectal disorders encountered in clinical practice and represent a substantial source of anorectal morbidity worldwide. They are characterized by the symptomatic enlargement and distal displacement of the normal anal cushions, leading to symptoms such as rectal bleeding, pain, pruritus, and discomfort during defecation^[^1–4^]^. Although hemorrhoidal cushions are normal anatomical structures that contribute to continence, pathological changes may lead to clinically significant symptoms requiring medical or surgical management^[^5^]^. Based on anatomical location, hemorrhoids are classified as internal and external; internal hemorrhoids arise above the dentate line and are covered by columnar epithelium, while external hemorrhoids arise below the dentate line and are covered by squamous epithelium^[^6–8^]^. Internal hemorrhoids can further be categorized into four degrees according to severity using the Goligher classification system: Grade I hemorrhoids bleed but do not prolapse; Grade II prolapse during straining but reduce spontaneously; Grade III prolapse and require manual reduction; and Grade IV are permanently prolapsed and irreducible^[^9,10^]^.

HIGHLIGHTS

- The prevalence of hemorrhoids was 6.1% among adult surgical inpatients at the Ali Abad Teaching Hospital.
- Rectal bleeding and anal pain were the most common presenting symptoms in affected patients.
- Constipation was the most frequently reported clinical and lifestyle-related factor among patients.
- Internal hemorrhoids were more common than external hemorrhoids, and advanced grades were frequently observed.
- The findings highlight the clinical characteristics and reported factors among patients with documented hemorrhoids.

The global prevalence of hemorrhoids has been reported to vary widely from 4.4 to 86%, depending on study design, population type (community-based versus hospital-based), and diagnostic criteria^[^11^]^, with the highest rates reported in Australia (38.93%), Saudi Arabia (30%), Egypt (18%), Israel (16%), Korea (14.4%), and Ethiopia (13.1%)^[^12–17^]^. Hemorrhoids are a leading cause of lower gastrointestinal bleeding and anorectal morbidity worldwide^[^18^]^. Although both sexes are affected and the condition may occur at any age, prevalence tends to increase with advancing age^[^19^]^. Several factors have been associated with the development of hemorrhoids. These include chronic constipation, prolonged straining during defecation, diarrhea, low-fiber diet, obesity, sedentary lifestyle, and pregnancy. Additional conditions such as prolonged sitting, chronic cough, spinal cord injury, and frequent Valsalva maneuvers may increase intra-abdominal pressure and impair venous return, thereby contributing to hemorrhoidal enlargement^[^20^]^. Socioeconomic and occupational factors may further influence disease occurrence, particularly in populations with limited health education and inadequate dietary fiber intake^[^21^]^. Beyond clinical symptoms, hemorrhoidal disease imposes considerable medical and socioeconomic burdens on affected individuals and healthcare systems. Patients frequently experience physical discomfort and limitations in daily activities that may reduce quality of life and work productivity^[^22^]^. Direct healthcare costs include medical consultations, medications, and procedures, while indirect costs arise from work absenteeism and productivity loss^[^23^]^.

In Afghanistan, epidemiological evidence regarding hemorrhoidal disease remains scarce and fragmented. To date, only one hospital-based study conducted in Kabul has examined the demographic characteristics of patients diagnosed with hemorrhoids. That study included 252 patients diagnosed between March 2021 and March 2022 and reported a mean age of 40.53 ± 15.08 years, a predominance of males (male-to-female ratio 5.15:1), and constipation as the most common comorbidity (34.9%)^[^24^]^. However, the investigation mainly described demographic characteristics and did not comprehensively evaluate the prevalence and clinical characteristics of hemorrhoids within a clearly defined inpatient population, thereby leaving a significant knowledge gap in the epidemiology of hemorrhoidal disease in Afghanistan. Therefore, this study aimed to determine the prevalence and describe the clinical characteristics of hemorrhoids among adult surgical inpatients at a public teaching hospital in Kabul, Afghanistan. By addressing this knowledge gap, the study provides important epidemiological evidence from a region where data on anorectal diseases remain scarce and contributes to the broader understanding of hemorrhoidal disease in low-resource settings.

## Methods

### Study design and setting

This study was designed as a retrospective cross-sectional study and was conducted at a public teaching hospital in Kabul, Afghanistan. The hospital is one of the major referral centers in the country and provides a wide range of surgical and medical services. It serves patients from various provinces and includes departments such as General Surgery, Internal Medicine, Orthopedics, Urology, Neurosurgery, and Radiology. The study utilized medical records from adult surgical inpatients documented between January and December of 2025.

### Sampling method and study population

The study employed a consecutive sampling method. All medical records of adult patients admitted to the General Surgery Department during the study period were screened for eligibility. Over the one-year study period, a total of 3781 patients were admitted to the General Surgery Department. After excluding two patients younger than 18 years, 3779 adult surgical admissions constituted the study population. Among these adult admissions, 229 patients had a clinically documented diagnosis of hemorrhoids, corresponding to a hospital-based prevalence of 6.1% (229/3779). The hospital-based prevalence was calculated by dividing the number of adult surgical admissions with a documented clinical diagnosis of hemorrhoids (229) by the total number of adult surgical admissions (3779). All adult surgical inpatients underwent a routine anorectal clinical examination by the attending surgical team during their admission, regardless of whether they reported anorectal symptoms. Hemorrhoid diagnoses identified during this assessment were documented in the medical record and included in the hospital-based prevalence estimate.

However, analyses of patient characteristics and clinical features of hemorrhoids were restricted to the 209 patients with complete medical records who met the eligibility criteria after applying the prespecified exclusion criteria. No formal sample size calculation was performed because this retrospective cross-sectional study used consecutive sampling and included all eligible patients with a documented diagnosis of hemorrhoids during the study period. Therefore, the final analytical sample represented the total available population that met the prespecified eligibility criteria.

### Data collection

Data were collected from the medical records of adult surgical inpatients admitted during the study period. As part of routine inpatient surgical care, patients were clinically assessed by the attending surgical team, including assessment for anorectal complaints and clinical findings. All adult surgical inpatients underwent a routine anorectal clinical examination during their admission, and hemorrhoid diagnoses identified clinically were documented in the medical record by the attending surgeon. This approach allowed identification of hemorrhoid diagnoses documented during patients’ inpatient admissions, including those identified during routine clinical assessments. Of the 231 documented hemorrhoid cases identified in the hospital records, 229 were adults, and 2 were younger than 18 years; only the 229 adult cases contributed to the hospital-based prevalence estimate. To ensure diagnostic accuracy, only records with a confirmed and clearly documented diagnosis of hemorrhoids and complete clinical information were included in the study. Cases with incomplete documentation or uncertainty regarding the diagnosis were excluded from the analysis. Data extraction was performed by trained researchers using a structured, pretested data extraction form. The form was developed to ensure consistent and systematic retrieval of relevant variables from patient records. Variables included sociodemographic characteristics (age group, sex, province of residence, occupation, marital status, and socioeconomic status), clinical characteristics (family history, comorbidities, signs, and symptoms), and disease-related characteristics (type and degree of hemorrhoids, reported clinical and lifestyle-related factors, and treatment modality). Prior to analysis, all records were reviewed to assess completeness and accuracy of the extracted information. Only patients with complete medical records were included in the final dataset. For the purposes of this study, hemorrhoids were categorized as internal, external, or mixed based on clinical documentation in patient records. Internal hemorrhoids were graded using the Goligher classification system (grades I–IV), while external hemorrhoids were recorded without grading. Mixed hemorrhoids were defined as the coexistence of internal and external components, as documented by the attending surgeon.

### Operational definitions

- Constipation was defined as a documented history or a clinical diagnosis of constipation recorded in the medical record by the treating clinical team.

- Prolonged sitting or standing was defined as a documented history of extended periods of sitting or standing related to occupational or daily activities, as recorded in the medical record.

- Socioeconomic status was categorized as lower, middle, or upper according to the socioeconomic classification documented in the patient’s medical record at the time of admission.

- Multiple vaginal deliveries were defined as a history of two or more vaginal deliveries documented in the medical record of female patients.

### Inclusion criteria and exclusion criteria

Patients were eligible for inclusion in the clinical analysis if they were aged 18–80 years, had a confirmed diagnosis of hemorrhoids, and had complete medical records. Of the 231 patients with a documented diagnosis of hemorrhoids, 2 were younger than 18 years and were therefore excluded from the adult study population. Among the remaining 229 adult patients with hemorrhoids, 20 were excluded because of incomplete medical records (*n* = 11), anorectal tumors (*n* = 4), or concomitant anorectal conditions such as fissure or fistula (*n* = 5). Consequently, 209 adult patients were included in the final clinical analysis (Fig. 1).

**Figure 1.**
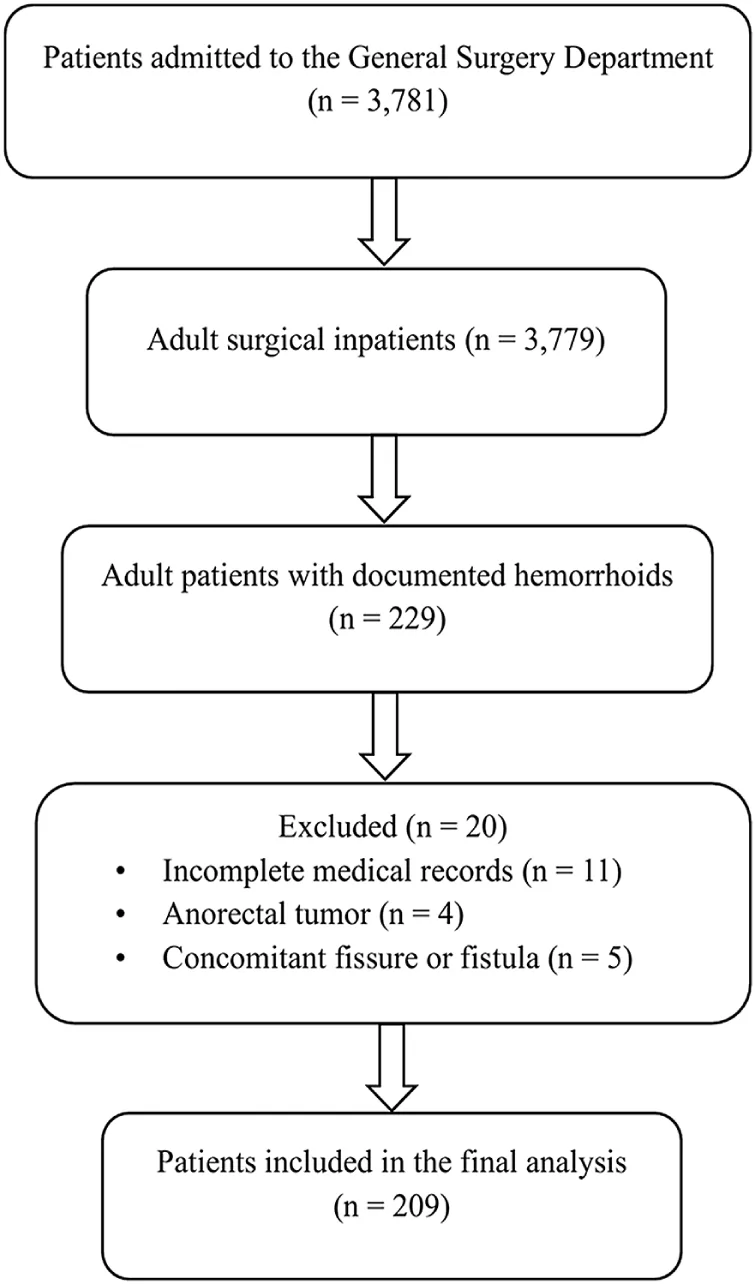
Flow diagram of patient selection. This diagram illustrates the patient selection process at Ali Abad Teaching Hospital, Kabul, Afghanistan, from January to December 2025. A total of 3,781 patients were admitted to the General Surgery Department. After excluding two patients younger than 18 years, 3,779 adult surgical inpatients constituted the study population. Among these adult admissions, 229 patients had a documented clinical diagnosis of hemorrhoids. After applying the predefined exclusion criteria, 20 patients were excluded because of incomplete medical records (n = 11), anorectal tumors (n = 4), or concomitant anorectal conditions such as fissure or fistula (n = 5). Consequently, 209 adult patients with hemorrhoids were included in the final clinical analysis.

### Statistical analysis

All statistical analyses were performed using IBM SPSS Statistics version 29 (IBM Corp., Armonk, NY, USA). Descriptive statistics were used to summarize the study findings. Categorical variables were presented as frequencies and percentages, while continuous variables were summarized as medians with interquartile ranges (IQRs) or means with standard deviations, as appropriate. The normality of continuous variables was assessed using the Shapiro–Wilk test. As age was not normally distributed according to the Shapiro–Wilk test, it was summarized using the median and IQR, and non-parametric methods were used for comparisons across groups. Associations between categorical variables, including hemorrhoid type, degree, and sociodemographic characteristics, were assessed using Pearson’s chi-square test. When expected cell counts were fewer than 5, the Fisher–Freeman–Halton exact test was used, as appropriate. Differences in age across hemorrhoid-degree groups were assessed using the Kruskal–Wallis test. A two-sided *P*-value < 0.05 was considered statistically significant.

## Results

A total of 3781 patients were admitted to the General Surgery Department during the study period, of whom 3779 were adults. Among the adult surgical inpatients, 229 had a clinically documented diagnosis of hemorrhoids, corresponding to a hospital-based prevalence of 6.1% (229/3779). After applying the predefined exclusion criteria, 20 patients were excluded, leaving 209 adult patients with hemorrhoids for the final clinical analysis (Fig. 1). The mean age of the patients was 49.07 ± 18.27 years, while the median age was 50 years (IQR: 33.5–65.0 years). Among the included patients, 128 (61.2%) were male, and 81 (38.8%) were female, resulting in a male-to-female ratio of 1.58:1. Most patients were married: 118 (56.5%). The highest number of analyzed hemorrhoid cases was observed in the 30–39-year age group, comprising 64 (30.6%) patients. Overall, patients aged 30–49 years accounted for more than half of the analyzed hemorrhoid cases. Regarding residence, a significant proportion of patients were from Kabul: 71 (34%), while other provinces contributed smaller numbers. By occupation, government employees represented the largest group: 82 (39.2%), followed by self-employed individuals: 47 (22.5%), unemployed individuals: 45 (21.5%), and housewives: 35 (16.7%). The socioeconomic status of patients was mainly middle class: 103 (49.3%), with lower- and upper-class individuals accounting for 69 (33%) and 37 (17.7%), respectively. A family history of hemorrhoids was reported by 72 (34.4%) patients. Regarding comorbidities, hypertension: 43 (20.6%), mental disorders: 40 (19.1%), diabetes: 33 (15.8%), and tuberculosis: 30 (14.4%) were the most commonly recorded medical conditions. These patient characteristics are summarized in Table 1.

**Table 1:** Sociodemographic characteristics of patients (*n* = 209).

Characteristics	Frequency	Percent (%)
Sex
Male	128	61.2
Female	81	38.8
Age group
18–29	37	17.7
30–39	64	30.6
40–49	51	24.4
50–59	23	11
60–69	21	10
70–80	13	6.2
Province of residence
Kabul	71	34
Balkh	14	6.7
Herat	13	6.2
Nangarhar	18	8.6
Baghlan	15	7.2
Kunar	16	7.7
Laghman	20	9.6
Parwan	14	6.7
Kapisa	15	7.2
Panjshir	13	6.2
Occupation
Unemployed	45	21.5
Government employee	82	39.2
Self-employed	47	22.5
Housewife	35	16.7
Marital status
Married	118	56.5
Divorced/Widowed	8	3.8
Single	83	39.7
Socioeconomic status
Lower	69	33
Middle	103	49.3
Upper	37	17.7
Family history of hemorrhoids
Yes	72	34.4
No	137	65.6
Comorbidities
Hypertension	43	20.6
Diabetes	33	15.8
TB	30	14.4
Mental disorders	40	19.1

Comorbidities were not mutually exclusive; therefore, patients could have more than one comorbidity, and the corresponding percentages do not necessarily sum to 100%.

The most frequently observed symptom among patients was rectal bleeding, 126 (60.3%), followed by anal pain, 115 (55%). Other less common complaints included anal lumps, 102 (48.8%), and itching or burning sensations, 88 (42.1%). These clinical characteristics are summarized in Fig. 2.

**Figure 2.**
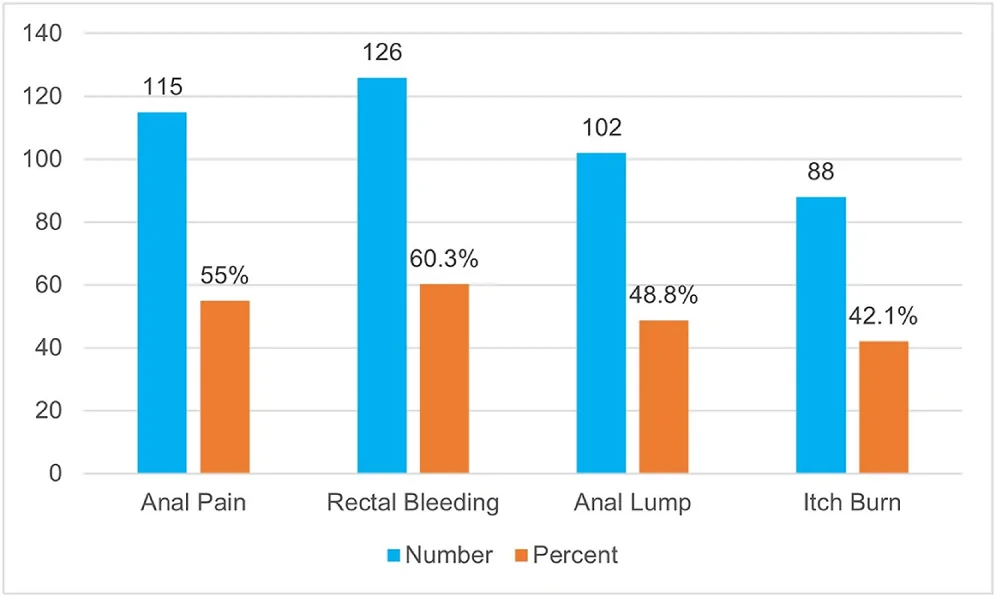
Distribution of reported symptoms among patients with hemorrhoids. Symptom categories were not mutually exclusive; therefore, percentages do not total 100%.

The most commonly reported factor among patients with hemorrhoids was constipation, affecting 111 (53.1%), followed by prolonged periods of standing or sitting, affecting 103 (49.3%). Among female patients, multiple vaginal deliveries were reported in 58 (71.6%) cases. These reported factors are summarized in Fig. 3.

**Figure 3.**
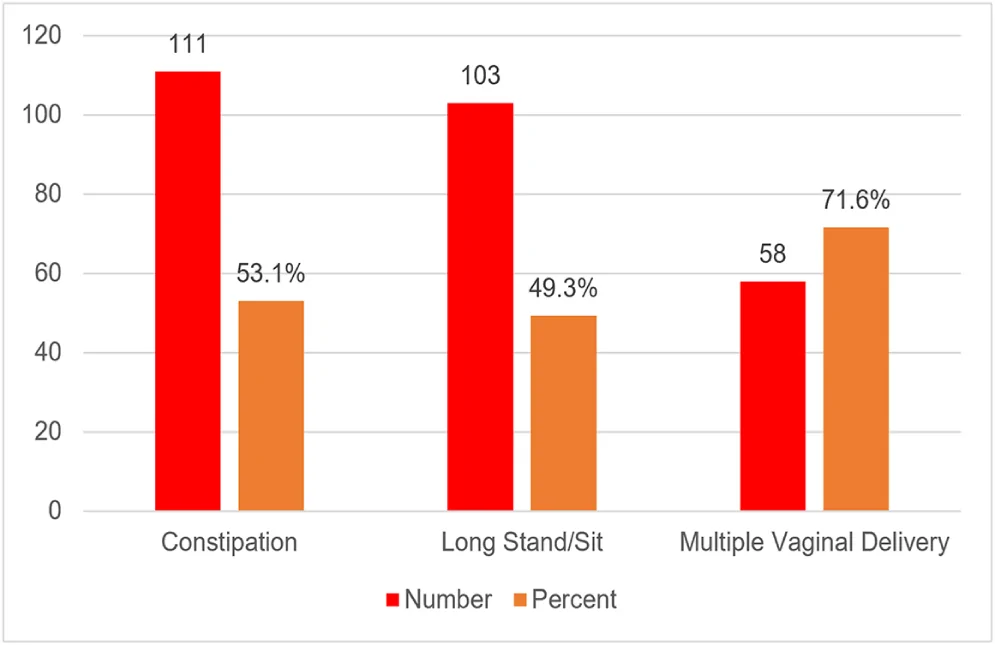
Distribution of reported factors among patients with hemorrhoids. Multiple vaginal deliveries were assessed only among female patients (n = 81); therefore, percentages for this variable were calculated using the female subgroup as the denominator.

Among the study population, external hemorrhoids were observed in 72 patients (34.4%). Regarding internal hemorrhoids, third-degree was the most frequently observed grade, with 32 (15.3%), followed by second-degree, 29 (13.9%); fourth-degree, 25 (12.0%); and first-degree, 20 (9.6%). In patients with mixed hemorrhoids, third-degree was also the most common, with 11 (5.3%), followed by second-degree, 10 (4.8%); first-degree, 7 (3.3%); and fourth-degree, 3 (1.4%). These distributions are summarized in Table 2. Although a higher proportion of males had external hemorrhoids, 50 (69.4%), compared with females, 22 (30.6%), the difference was not statistically significant (*P* = 0.203). Similarly, no significant differences were observed across age groups or by family history of hemorrhoids. Notably, a statistically significant difference was observed in the distribution of hemorrhoid types across marital-status groups (*P* = 0.028). However, this finding should be interpreted as an exploratory difference in distributions, particularly given the small number of divorced or widowed patients. The distribution of hemorrhoid types across selected patient characteristics is presented in Table 3.

**Table 2:** Distribution of hemorrhoid types and grades among patients with hemorrhoids (*n* = 209).

Types of Hemorrhoids	Degree	Number of Patients	Percent (%)
External hemorrhoid	-	72	34.4
Internal hemorrhoid	First degree	20	9.6
	Second degree	29	13.9
	Third degree	32	15.3
	Fourth degree	25	12.0
Mixed	First degree	7	3.3
	Second degree	10	4.8
	Third degree	11	5.3
	Fourth degree	3	1.4

All percentages are calculated based on the total number of patients with hemorrhoids (*n* = 209) to ensure consistency and comparability across all categories.

**Table 3:** Association between hemorrhoid type and sociodemographic characteristics.

Variable	External, n (%)	Internal, n (%)	Mixed, *n* (%)	*P*-value
Sex				
Male	50 (69.4%)	61 (57.5%)	17 (54.8%)	0.203
Female	22 (30.6%)	45 (42.5%)	14 (45.2%)	
Age group				
18–29	15 (20.8%)	17 (16%)	5 (16.1%)	0.410
30–39	23 (31.9%)	31 (29.2%)	10 (32.3%)	
40–49	12 (16.7%)	31 (29.2%)	8 (25.8%)	
50–59	6 (8.3%)	12 (11.3%)	5 (16.1%)	
60–69	12 (16.7%)	7 (6.6%)	2 (6.5%)	
70–80	4 (5.6%)	8 (7.5%)	1 (3.2%)	
Marital status				
Married	39 (54.2%)	63 (59.4%)	16 (51.6%)	0.028
Single	33 (45.8%)	39 (36.8%)	11 (35.5%)	
Divorced/widowed	0	4 (3.8%)	4 (12.9%)	
Family history of hemorrhoids				
Yes	30 (41.7%)	36 (34%)	6 (19.4%)	0.091
No	42 (58.3%)	70 (66%)	25 (80.6%)	
Constipation				
Yes	34 (47.2%)	64 (60.4%)	13 (41.9%)	0.091
No	38 (52.8%)	42 (39.6%)	18 (58.1%)	

Associations were assessed using the Chi-square test. Fisher–Freeman–Halton exact test was used when expected cell counts were less than 5. A *P*-value < 0.05 was considered statistically significant.

The association between Goligher grade and selected patient characteristics among patients with internal or mixed hemorrhoids is presented in Table 4. The median age increased from 41.0 years (Q1–Q3: 27.0–61.0) among patients with Degree I hemorrhoids to 56.5 years (Q1–Q3: 39.0–67.0) among those with Degree IV; however, the difference in age across hemorrhoid grades was not statistically significant (*P* = 0.323, Kruskal–Wallis test). Similarly, sex distribution did not differ significantly across hemorrhoid grades (*P* = 0.198, Pearson’s chi-square test). Males comprised 63.0, 48.7, 67.4, and 46.4% of patients with Degrees I, II, III, and IV, respectively, while females comprised 37.0, 51.3, 32.6, and 53.6%, respectively. Family history of hemorrhoids also showed no statistically significant association with hemorrhoid degree (*P* = 0.246, Pearson’s chi-square test), with a positive family history reported by 29.6, 41.0, 30.2, and 17.9% of patients with Degrees I, II, III, and IV, respectively.

**Table 4:** Association between Goligher grade and selected patient characteristics among patients with internal or mixed hemorrhoids (*n* = 137).

Variable	Degree I, *n* (%)	Degree II, *n* (%)	Degree III, *n* (%)	Degree IV, *n* (%)	*P*-value
Age (years)	41.0 (27.0–61.0)	53.0 (33.5–61.5)	53.0 (42.0–66.5)	56.5 (39.0–67.0)	0.323*
Sex					
Male	17 (63%)	19 (48.7%)	29 (67.4%)	13 (46.4%)	0.198**
Female	10 (37%)	20 (51.3%)	14 (32.6%)	15 (53.6%)	
Family history of hemorrhoids					
Yes	8 (29.6%)	16 (41%)	13 (30.2%)	5 (17.9%)	0.246**
No	19 (70.4%)	23 (59%)	30 (69.8%)	23 (82.1%)	

The grade analysis included only patients with internal or mixed hemorrhoids (*n* = 137), as external hemorrhoids were not graded. Age is presented as median (Q1–Q3).

^*^ *P*-value was calculated using the Kruskal–Wallis test.

^**^ *P*-values were calculated using Pearson’s chi-square test

## Discussion

In this hospital-based study of adult surgical inpatients at a public teaching hospital in Kabul, clinically documented hemorrhoids were identified in 6.1% of admissions (229/3779), representing a hospital-based prevalence of 6.1% in this setting. Among the analyzed cases, the mean age was 49.07 years, and 61.2% were male. Internal hemorrhoids were the most common type, and constipation was the most frequently reported associated factor among patients with hemorrhoids. These findings provide important insight into the clinical and demographic characteristics of patients with documented hemorrhoids in this hospital-based surgical population.

The hospital-based prevalence of clinically documented hemorrhoids in this study was 6.1% among adult surgical admissions. This estimate was lower than those reported in several previous studies conducted in different countries, including Australia (38.9%)^[^12^]^, Egypt (18%)^[^14^]^, Israel (16%)^[^15^]^, South Korea (14.4%)^[^16^]^, Northwest Ethiopia (13.1%)^[^17^]^, and Saudi Arabia (13.29%)^[^25^]^. However, a considerably lower prevalence of 4.4% was reported by Johanson and Sonnenberg^[^26^]^. The variation in prevalence across studies may be attributed to differences in study design, population characteristics, diagnostic methods, and healthcare-seeking behavior. Furthermore, the present study was conducted among hospitalized surgical patients rather than the general population, which may partly explain the comparatively lower prevalence observed. However, this estimate should be interpreted as a hospital-based prevalence among adult surgical inpatients and should not be extrapolated to the general population. Comparisons with community-based prevalence estimates should therefore be made cautiously because differences in study populations, healthcare settings, diagnostic approaches, and case ascertainment may substantially influence the reported estimates.

Among the analyzed hemorrhoid cases, the largest proportion was observed among individuals aged 30–39 years, while patients aged 30–49 years accounted for more than half of the analyzed cases. Similar age distributions have been reported in studies conducted in Saudi Arabia^[^27^]^, where hemorrhoids were more commonly observed among individuals aged 18–40 years, and in India^[^19^]^, where the condition was frequently reported among individuals aged 20–39 years. Additionally, another study from India reported a higher occurrence of hemorrhoids among individuals aged 40 years and older^[^22^]^. Likewise, studies conducted in India and Afghanistan have also indicated that hemorrhoids are commonly observed among individuals within the age range of 20–40 years, or approximately 30–40 years^[^20,24^]^. These similarities in the age distribution of reported cases may reflect differences in lifestyle patterns, occupational conditions involving prolonged sitting or straining, dietary habits, and constipation; however, the present study cannot determine whether these factors increase the risk of hemorrhoids because age-specific denominators and a non-hemorrhoid comparison group were unavailable.

Among the analyzed hemorrhoid cases, males constituted 61.2% and females 38.8%. This male predominance in the distribution of documented cases is consistent with findings from previous studies conducted in Afghanistan, which reported a higher proportion of male cases (83.7 vs. 16.3%)^[^24^]^, and in India, where males were also more frequently affected than females (66.7 vs. 33.3% and 55 vs. 45%)^[^19,20^]^. Conversely, a study conducted in Saudi Arabia reported a higher prevalence among females (61.6 vs. 38.4%)^[^27^]^. These differences in sex distribution across studies may reflect variations in study populations, occupational exposures, lifestyle behaviors, healthcare-seeking patterns, and case ascertainment. However, because the sex distribution of all adult surgical admissions was not available, the present study cannot determine whether the occurrence or prevalence of hemorrhoids differed between males and females.

Constipation emerged as the most frequently reported factor among patients with hemorrhoids in this study, affecting 53.1% of patients. This finding is in line with previous research conducted in Afghanistan, where constipation was reported in 34.9% of cases as a risk factor^[^24^]^. Similar findings have also been reported in other studies^[^22,28^]^. Constipation may contribute to increased straining during defecation, which elevates intra-abdominal pressure and promotes venous congestion in the hemorrhoidal plexus, thereby facilitating the development or progression of hemorrhoids. Differences in the reported prevalence of constipation across studies may also reflect variations in dietary fiber intake, fluid consumption, physical activity, and other lifestyle factors among the populations studied.

Regarding sociodemographic characteristics, the majority of patients in this study resided in Kabul (34%) and were predominantly government employees (39.2%). In contrast, a previous study conducted in Afghanistan reported that most patients also lived in Kabul (69.5%) but were largely unemployed (57.1%)^[^24^]^. The observed differences in occupational distribution between studies may be related to variations in the study population, socioeconomic status, and patterns of healthcare access. Government employees may have different occupational and behavioral characteristics, such as prolonged sitting and reduced physical activity; however, the present study did not assess whether these characteristics were associated with hemorrhoid occurrence. In addition, a statistically significant difference was observed in the distribution of hemorrhoid types across marital-status groups (*P* = 0.028); however, this finding should be interpreted cautiously as an exploratory difference in distributions rather than evidence that married patients were more likely to have internal hemorrhoids. The small number of divorced or widowed patients may have contributed to the observed overall difference. No established biological mechanism explains this observation, and it may be influenced by unmeasured confounding factors or the effect of multiple testing. Therefore, this result should not be interpreted as a true causal or independent association and requires confirmation in future studies.

Analysis of clinical symptoms revealed that several complaints were commonly reported among patients with hemorrhoids. The most commonly reported symptoms were rectal bleeding (60.3%), anal pain (55%), anal lump (48.8%), and itching or burning sensation (42.1%). These findings are consistent with previous studies conducted in Afghanistan and India^[^19,24^]^, which reported similar patterns of symptom presentation. However, our findings differ from those reported in a study conducted in Iran, where pruritus (45.8%) was the most frequently reported symptom, followed by discharge (41.6%) and anal pain (22.5%)^[^3^]^. Such differences may be explained by variations in patient populations, clinical presentation, and healthcare-seeking behavior, as well as differences in disease severity at the time of presentation.

The distribution of hemorrhoid types in the present study showed that the majority of patients had internal hemorrhoids (50.7%). Among these, third- and fourth-degree internal hemorrhoids were predominant, often necessitating surgical intervention. This observation is consistent with a previous study conducted in Afghanistan^[^24^]^, which reported that 52% of patients had internal hemorrhoids, most of which were third- and fourth-degree, as well as with a study in India^[^29^]^, where advanced grades of internal hemorrhoids were also predominant. The higher proportion of advanced hemorrhoid grades observed in the present study may be related to the fact that the study population consisted of surgical inpatients, who are more likely to present with severe or complicated forms of the disease that require hospital admission and surgical management.

Overall, the findings of this study contribute to a better understanding of the epidemiological and clinical characteristics of hemorrhoidal disease among hospitalized patients in Kabul. The frequent occurrence of constipation among patients with hemorrhoids highlights the importance of preventive strategies focusing on dietary modification, adequate fluid intake, increased physical activity, and lifestyle changes. Furthermore, improving public awareness regarding early symptoms and modifiable risk factors may facilitate earlier diagnosis and management of hemorrhoids, thereby potentially reducing disease complications and the need for surgical intervention.

### Limitations

This study has several limitations that should be acknowledged. First, the retrospective design relied on medical records, which may have resulted in incomplete or missing information for some variables and may have introduced information bias. Second, only patients with complete medical records were included in the final analysis, which may have introduced selection bias. Third, the study was conducted in a single tertiary hospital and included only surgical inpatients; therefore, the reported prevalence may not be generalizable to the general population or to non-surgical patient populations. Fourth, because the study included only patients diagnosed with hemorrhoids and did not include a comparison group without hemorrhoids, it was not possible to identify independent risk factors or perform multivariable analyses to assess factors associated with disease occurrence. Therefore, the reported factors should be interpreted as characteristics observed among patients with hemorrhoids rather than as causal or independently associated factors. In addition, multiple statistical comparisons were performed in the exploratory analyses without adjustment for multiple testing. Therefore, the possibility of Type I error cannot be excluded, and statistically significant findings should be interpreted with caution. Despite these limitations, the study provides valuable epidemiological insights into the prevalence and clinical characteristics of hemorrhoids in a hospital-based surgical population in Kabul, Afghanistan.

## Conclusion

This study provides valuable epidemiological insights into the prevalence and clinical characteristics of hemorrhoids among adult surgical inpatients at a public teaching hospital. Clinically documented hemorrhoids were identified in 6.1% of adult surgical admissions, representing a hospital-based prevalence in this surgical inpatient population. The most frequently reported symptoms were rectal bleeding and anal pain, whereas constipation was the most frequently reported modifiable factor among patients with hemorrhoids. Internal hemorrhoids, particularly higher-grade forms requiring surgical management, were predominant among the study population. These findings highlight the clinical characteristics of hemorrhoids and emphasize the importance of early recognition and management of commonly reported factors such as constipation and lifestyle-related behaviors among patients with hemorrhoids. Implementing targeted preventive strategies, enhancing patient education, and promoting timely clinical evaluation may contribute to reducing the burden, complications, and healthcare costs associated with hemorrhoids in similar hospital settings.

## Data Availability

The data are not publicly available due to ethical restrictions and anonymity but can be obtained upon request from the corresponding author via email: bashirqudrati786@gmail.com.
